# Diversity of rotavirus genotypes circulating in children < 5 years of age hospitalized for acute gastroenteritis in India from 2005 to 2016: analysis of temporal and regional genotype variation

**DOI:** 10.1186/s12879-020-05448-y

**Published:** 2020-10-09

**Authors:** Sidhartha Giri, C. P. Girish Kumar, Shainey Alokit Khakha, Mamta Chawla-Sarkar, Varanasi Gopalkrishna, Shobha D. Chitambar, Pratima Ray, S. Venkatasubramanian, Biswa Jyoti Borkakoty, Subarna Roy, Jyothi Bhat, Bhagirathi Dwibedi, Pradeep Das, Vijayachari Paluru, Sasirekha Ramani, Sudhir Babji, Rashmi Arora, Sanjay M. Mehendale, Mohan D. Gupte, Gagandeep Kang, National Rotavirus Surveillance Network investigators, A. Agarwal, S. Aneja, Anna Simon, S. C. Aundhakar, A. Bavdekar, S. Baveja, D. Biswas, C. J. Bora, S. Chatterjee, S. Chaudhary, Das VNR, K. Desai, R. Dhongade, R. Dwivedi, K. Dzuvichu, N. Ganguly, G. Gathwala, C. Ghosh, D. S. Gupta, A. R. Jadhav, S. Jali, V. R. Kalrao, S. K. Kar, H. K. Khuntia, P. Kumar, S. S. Kumar, B. G. Lal, M. Manglani, B. Manohar, A. Mathew, M. A. Mathew, K. M. Mehariya, S. K. Mishra, S. Panda, K. Pandey, M. Patankar, C. S. Purani, G. C. Sahoo, N. Singh, P. Singh, T. Singh, S. Sundari, A. K. Thakur, R. K. Topno, A. Upadhyay, Utpalkant Singh

**Affiliations:** 1 Division of Gastrointestinal Sciences, Christian Medical College, Vellore, Tamil Nadu India; 2 Indian Council of Medical Research, New Delhi, India; 3 National Institute of Epidemiology, Chennai, Tamil Nadu India; 4 National Institute of Cholera and Enteric Diseases, Kolkata, West Bengal India; 5 National Institute of Virology, Pune, Maharashtra India; 6 Jamia Hamdard, New Delhi, India; 7 Regional Medical Research Centre, Dibrugarh, Assam India; 8 National Institute of Traditional Medicine, Belgaum, Karnataka India; 9 National Institute for Research in Tribal Health, Jabalpur, Madhya Pradesh India; 10 Regional Medical Research Centre, Bhubaneswar, Odisha India; 11 Rajendra Memorial Research Institute of Medical Sciences, Patna, Bihar India; 12 Regional Medical Research Centre, Port Blair, Andaman & Nicobar Islands India; 13 Baylor College of Medicine, Houston, TX USA; 14 Translational Health Science and Technology Institute (THSTI), Faridabad, Haryana India

**Keywords:** Diarrhoea, Enzyme immunoassay, Gastroenteritis, Genotypes, India, Polymerase chain reaction, Rotavirus

## Abstract

**Background:**

From 2016, the Government of India introduced the oral rotavirus vaccine into the national immunization schedule. Currently, two indigenously developed vaccines (ROTAVAC, Bharat Biotech; ROTASIIL, Serum Institute of India) are included in the Indian immunization program. We report the rotavirus disease burden and the diversity of rotavirus genotypes from 2005 to 2016 in a multi-centric surveillance study before the introduction of vaccines.

**Methods:**

A total of 29,561 stool samples collected from 2005 to 2016 (7 sites during 2005–2009, 3 sites from 2009 to 2012, and 28 sites during 2012–2016) were included in the analysis. Stools were tested for rotavirus antigen using enzyme immunoassay (EIA). Genotyping was performed on 65.8% of the EIA positive samples using reverse transcription- polymerase chain reaction (RT-PCR) to identify the G (VP7) and P (VP4) types. Multinomial logistic regression was used to quantify the odds of detecting genotypes across the surveillance period and in particular age groups.

**Results:**

Of the 29,561 samples tested, 10,959 (37.1%) were positive for rotavirus. There was a peak in rotavirus positivity during December to February across all sites. Of the 7215 genotyped samples, G1P[8] (38.7%) was the most common, followed by G2P[4] (12.3%), G9P[4] (5.8%), G12P[6] (4.2%), G9P[8] (4%), and G12P[8] (2.4%). Globally, G9P[4] and G12P[6] are less common genotypes, although these genotypes have been reported from India and few other countries. There was a variation in the geographic and temporal distribution of genotypes, and the emergence or re-emergence of new genotypes such as G3P[8] was seen. Over the surveillance period, there was a decline in the proportion of G2P[4], and an increase in the proportion of G9P[4]. A higher proportion of mixed and partially typed/untyped samples was also seen more in the age group 0–11 months.

**Conclusions:**

This 11 years surveillance highlights the high burden of severe rotavirus gastroenteritis in Indian children < 5 years of age before inclusion of rotavirus vaccines in the national programme. Regional variations in rotavirus epidemiology were seen, including the emergence of G3P[8] in the latter part of the surveillance. Having pre-introduction data is important to track changing epidemiology of rotaviruses, particularly following vaccine introduction.

## Background

Rotavirus has been a major cause of mortality among children under 5 years old, with approximately 128,500 deaths globally [1, 2]. Rotavirus gastroenteritis is a major cause of hospitalization in children < 5 years in India, responsible for 11.37 million episodes of acute gastroenteritis each year, requiring 3.27 million outpatient visits and 872,000 hospitalizations, accounting for Indian Rupee (INR) 10.37 billion per year in direct costs [3]. According to estimates from 2011 to 2013, rotavirus gastroenteritis caused approximately 78,000 deaths annually in India, of which 59,000 occur in children < 2 years of age [3]. The proportion of diarrhoea cases due to rotavirus has increased approximately from 25% (inter study variation [ISV]: 21–28%) in studies conducted before 2000, to more than 38% (ISV: 19–50%) in studies completed after 2005 [4]. A substantial diversity of rotavirus genotypes causing acute watery diarrhoea in the under five age group has been reported from surveillance studies on rotavirus gastroenteritis in India [5–12].

Since 2006, two oral rotavirus vaccines, Rotarix (monovalent G1P[8]; GlaxoSmithKline Biologicals, Belgium) and RotaTeq (pentavalent G1, G2, G3, G4, P[8]; Merck Vaccines, NJ, USA), have been commercially available in India only in the private market, and the coverage was less than 1% [4]. Two indigenously developed live oral rotavirus vaccines, ROTAVAC (Bharat Biotech, India) and ROTASIIL (Serum Institute of India, Pune, India) have been licensed in India [13, 14]. ROTAVAC (Bharat Biotech) containing the 116E rotavirus strain (G9P[11]), was licensed in 2014, and obtained the World Health Organization (WHO) prequalification in January 2018 [14]. ROTASIIL (Serum Institute of India), which contains G1, G2, G3, G4, and G9 (bovine-human reassortant pentavalent vaccine), was licensed in 2016, and was pre-qualified in late 2018 [14]. Based on the recommendation by the National Technical Advisory Group for Immunization (NTAGI), the Ministry of Health and Family Welfare in India approved the introduction of the oral rotavirus vaccine into the national immunization schedule in 2015 [14]. During 2016 to 2017, ROTAVAC was introduced in 9 Indian states, covering > 35% of the Indian birth cohort, while ROTASIIL was introduced into the immunization schedule in one state in 2018 [14]. Subsequently, all states have been covered, with about 60% of the population receiving ROTAVAC and 40% ROTASIIL.

Pre- vaccine surveillance data on the epidemiology of rotavirus gastroenteritis is crucial to understand any shifting trends after vaccine introduction. We report the findings from different phases of a national multicentre hospital-based surveillance on rotavirus gastroenteritis in children < 5 years from 2005 to 2016, focussing on the diversity, temporal and regional variation of circulating rotavirus genotypes.

## Methods

### Study sites

**Fig. 1:**
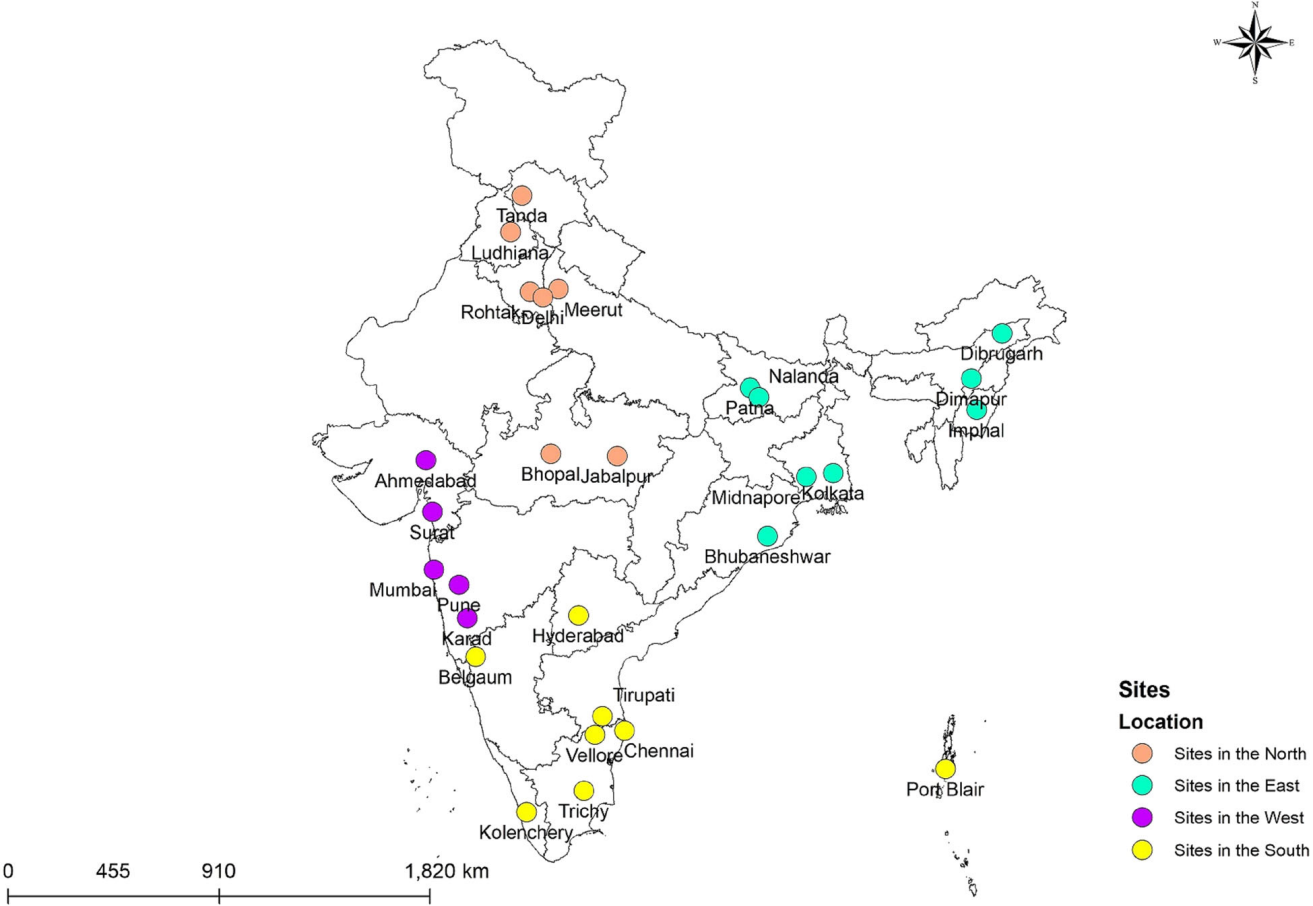
Sites in the rotavirus strain surveillance network in India from 2005 to 2016. 7 sites during 2005–2009, 3 sites during 2009 to 2012, 28 sites during 2012 to 2016. The map was created using ArcGIS® software by Esri. ArcGIS® and ArcMap™ are the intellectual property of Esri and are used herein under license. Copyright© Esri. All rights reserved. For more information about Esri® software, please visit www.esri.com

During November 2005 to June 2009, 10 hospitals from 7 Indian cities were included in the Indian Rotavirus Strain Surveillance Network, with testing for rotavirus being performed at 4 regional laboratories (Kolkata, Pune, Mumbai, Vellore) [15]. The study used a modification of the World Health Organization (WHO) generic protocol for rotavirus surveillance, and was supervised by the Indian Council of Medical Research (ICMR) and the Centres for Disease Control and Prevention (CDC, Atlanta) [16]. During July 2009 to June 2012, 3 hospitals associated with the Vellore regional laboratory continued the rotavirus surveillance [17]. From July 2012 to August 2016, multi-centric hospital based surveillance was conducted at 28 sites in India, with 4 referral centres for testing (Kolkata, Delhi, Pune, Vellore) (Fig. 1). The 4 year study was coordinated by the ICMR, the Christian Medical College (CMC), Vellore and the National Institute of Epidemiology (NIE), Chennai.

These multi-centric surveillance data combine the results of the earlier two surveillance studies on rotavirus gastroenteritis in Indian children < 5 years (2005–2009, 2009–2012) with that of 4 years of surveillance from 2012 to 2016 in up to 28 sites across India to provide a description of the overall distribution and diversity of rotavirus genotypes before the introduction of the oral rotavirus vaccine into the national immunization schedule [15, 17]. All the surveillance studies had the same enrolment criteria, and methods of testing, which included initial screening of stool samples for rotavirus by enzyme immunoassay (EIA), and genotyping of EIA positive samples.

### Enrolment criteria

Children aged ≤59 months of age hospitalized for acute gastroenteritis (AGE) for at least 6 h, and treated with oral and/or intravenous rehydration, were eligible for enrolment. An episode of AGE was defined as ≥3 episodes of watery stool within a 24 h period. Eligible children were recruited after obtaining written informed consent from the parent/guardian. Stool samples were collected from the recruited children within 48 h of admission to hospital to rule out nosocomial infection. Children ≥60 months of age and those presenting with dysentery (blood in stool) were excluded from the study.

### Sample collection, storage, and transport

The stool samples were either transported to the testing laboratory within 2 h or stored at 4 °C at the site. The samples which were stored at 4 °C at the sites were transported to the testing laboratory every month in boxes with ice packs. On reaching the testing laboratory, samples were aliquoted, and tested for rotavirus antigen. The aliquots were then stored at -70 °C for further testing.

### Laboratory procedures

Stool samples were screened for rotavirus antigen at the testing laboratories using commercial enzyme immunoassay (EIA) kits recommended by the WHO (IDEIA Rotavirus kit, Oxoid; or Premier Rotaclone, Meridian Bioscience). 65.8% of the EIA positive samples were genotyped to identify the VP7 (G type) and VP4 (P type) genes using reverse transcription polymerase chain reaction (RT-PCR) assays following published protocols [15, 18–20]. Prior to the RT-PCR assays, viral RNA was extracted from 20% stool suspension, followed by reverse transcription to generate complementary DNA (cDNA) using random primers (Invitrogen) and Moloney murine reverse transcriptase enzyme (Superscript II MMLV-RT, Invitrogen). The cDNA was then used in hemi-nested multiplex RT-PCR assays to identify the G type (G1, G2, G3, G4, G8, G9, G10, G12) and P type (P[4], P[6], P[8], P[9], P[10], P[11]) using published oligonucleotide primers [15, 18–20]. For samples negative for G type (VP7) and P type (VP4), a VP6 gene specific PCR was performed to confirm rotavirus positivity [15, 18]. The study protocol included quality assurance by testing of blinded samples exchanged between laboratories for rotavirus antigen by EIA and genotyping of EIA positive samples. Sanger sequencing was performed to confirm the unusual rotavirus genotypes such as G1P[4], G1P[6], G2P[6], G2P[8], G3P[6], G4P[6], G9P[6], G10P[11], G12[P4], and G12P[11].

### Statistical analysis

All sites submitted completed case report forms on all participants recruited in the surveillance. All the case report forms were scrutinized for completeness. The clinical and laboratory data were entered in Excel 2003 (Microsoft), and were analyzed to evaluate the proportion of rotavirus associated diarrhoea, genotype diversity (G and P types), temporal and regional variation in rotavirus genotypes across the four geographical zones (north, south, east, west) from 2005 to 2016. To evaluate the prevalence of rotavirus associated diarrhoea across the four regions, the proportion of diarrhoeal stool samples positive for rotavirus was calculated by region. To evaluate the regional variation in rotavirus positivity from 2005 to 2016, the monthly proportion of rotavirus positivity by EIA in the four zones (north, south, east, west) were compared.

We fitted mixed effect multinomial logistic regression models with genotype as the outcome variable and G1P[8] as the reference genotype. We included the year of surveillance, region (north, south, east, west), and age groups as independent variables in the model. As outcomes, we included the G1P[8], G2P[4] and G9P[4] genotypes, each of which had an overall proportion of > 5% among genotyped samples. In the outcome variable, the less common genotypes were grouped under “others”. In addition, mixed and untyped/partially typed samples were also included as separate categories in the regression analysis. Age was categorized into three groups; 0–11 months, 12–23 months, and 24–59 months. All statistical analysis was performed using IBM SPSS Statistics for Windows, version 21 (IBM Corp., Armonk, N.Y., USA). A p- value of < 0.05 was considered statistically significant.

### Ethics

The study was approved by the institutional review boards (ethics committees) of Christian Medical College (CMC, Vellore), National Institute of Virology (NIV, Pune), National Institute of Cholera and Enteric Diseases (NICED, Kolkata), All India Institute of Medical Sciences (AIIMS, New Delhi), and the site specific ethics committees associated with each hospital.

## Results

**Fig. 2:**
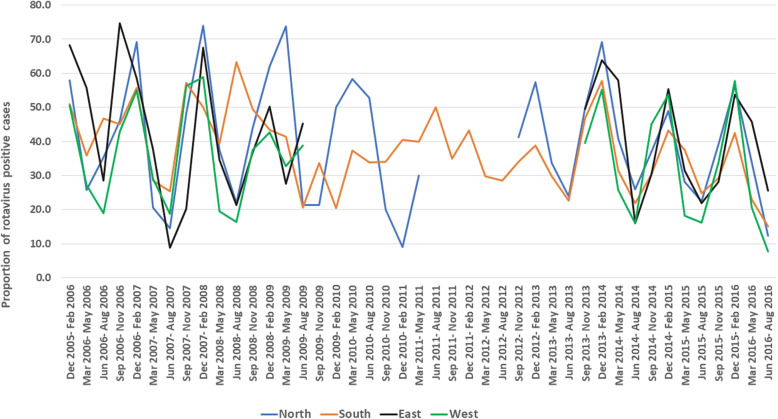
Temporal distribution of rotavirus-positive cases in the four geographical regions of India

From November 2005 through June 2016, stool samples collected from 29,561 enrolled children were tested for rotavirus. Overall, 10,959 (37.1%) samples were positive for rotavirus (Table 1). Rotavirus associated diarrhoea was seen throughout the year in all geographical regions although there was a difference in the year-wise positivity between the regions. The peak positivity rates were noted between December–February in all four regions (Fig. 2).

**Table 1:** Stool samples tested by EIA and positive for rotavirus by region from December 2005 to August 2016

Time period	North		South		East		West		Total	
	Number of samples	Rotavirus positive samples (%)	Number of samples	Rotavirus positive samples (%)	Number of samples	Rotavirus positive samples (%)	Number of samples	Rotavirus positive samples (%)	Number of samples	Rotavirus positive samples (%)
**December, 2005- August, 2006**	189	71 (37.6%)	502	227 (45.2%)	122	64 (52.5%)	462	151 (32.7%)	1275	513 (40.2%)
**September, 2006- August, 2007**	279	85 (30.5%)	492	184 (37.4%)	199	100 (50.3%)	730	265 (36.3%)	1700	634 (37.3%)
**September, 2007- August, 2008**	260	98 (37.7%)	425	226 (53.2%)	663	271 (40.9%)	700	250 (35.7%)	2048	845 (41.3%)
**September, 2008- August, 2009**	243	104 (42.8%)	361	137 (38%)	851	334 (39.2%)	661	250 (37.8%)	2116	825 (39%)
**September, 2009- August, 2010**	161	67 (41.6%)	312	103 (33%)	–	–	–	–	473	170 (35.9%)
**September, 2010- August, 2011**	46	10 (21.7%)	199	83 (41.7%)	–	–	–	–	245	93 (38%)
**September, 2011- August, 2012**	–	–	283	96 (33.9%)	–	–	–	–	283	96 (33.9%)
**September, 2012- August, 2013**	393	144 (36.6%)	1124	346 (30.8%)	–	–	–	–	1517	490 (32.3%)
**September, 2013- August, 2014**	1408	633 (45%)	1906	757 (39.7%)	1373	616 (44.9%)	1296	449 (34.6%)	5983	2455 (41%)
**September, 2014- August, 2015**	1630	541 (33.2%)	2306	783 (34%)	2732	1011 (37%)	1310	448 (34.2%)	7978	2783 (34.9%)
**September, 2015- August, 2016**	1040	366 (35.2%)	2058	613 (29.8%)	1741	701 (40.3%)	1104	375 (34%)	5943	2055 (34.6%)
**Total**	5649	2119 (37.5%)	9968	3555 (35.7%)	7681	3097 (40.3%)	6263	2188 (34.9%)	29,561	10,959 (37.1%)

Of the 10,959 samples positive for rotavirus by EIA, 7215 (65.8%) were genotyped. During 2005 to 2012, all the EIA positive samples were genotyped. For the surveillance from 2012 to 2016 involving 28 sites, the study protocol required that every third EIA positive sample be genotyped at the four reference laboratories. However, the reference laboratory at Vellore genotyped all the EIA positive samples as the site prepared and provided panels of genotyped samples for quality assurance. Hence, an overall of 65.8% (7215/ 10,959) samples were genotyped during the entire surveillance period in India from 2005 to 2016. Table 2 presents the distribution of genotypes, with G1P[8] (38.7%, 2789/7215) as the most common genotype, followed by G2P[4] (12.3%, 884/7215), G9P[4] (5.8%, 417/7215), G12P[6] (4.2%, 302/7215), and G9P[8] (4%, 287/7125). Uncommon rotavirus genotypes contributed to approximately 4.7% (340/7215) of the total genotyped samples. Mixed rotavirus genotypes (more than one G and/or P types) were seen in 6.7% (486/7215) of the genotyped samples (ranged from 5.2% in the south to 9.1% in the north Indian sites). Partially typed (either G or P typed) and untyped (neither G nor P typed) were seen in 8.6% (620/7215) and 10% (722/7215) samples respectively (Table 2). Partially typed and untyped samples were most common in the eastern region (14.6 and 27.5% respectively). The lowest proportion of partially typed and untyped samples were detected in the southern (5.5%) and western (3.3%) regions, respectively.

**Table 2:** Rotavirus genotype distribution (G and P types) in India from December 2005 to August 2016

Genotype	North		South		East		West		Total	
	N	%	N	%	N	%	N	%	N	%
**G1P[4]**	7	0.5	17	0.6	8	0.6	5	0.3	37	0.5
**G1P[6]**	47	3.6	36	1.2	10	0.7	37	2.4	130	1.8
**G1P[8]**	357	27.2	1413	47.7	409	28.6	610	40.3	2789	38.7
**G1P[9]**	1	0.1	0	0.0	1	0.1	0	0.0	2	0.0
**G1P[11]**	1	0.1	0	0.0	1	0.1	0	0.0	2	0.0
**G2P[4]**	150	11.4	394	13.3	84	5.9	256	16.9	884	12.3
**G2P[6]**	23	1.8	8	0.3	25	1.7	14	0.9	70	1.0
**G2P[8]**	3	0.2	6	0.2	8	0.6	15	1.0	32	0.4
**G2P[10]**	0	0.0	0	0.0	0	0.0	2	0.1	2	0.0
**G2P[11]**	1	0.1	0	0.0	1	0.1	0	0.0	2	0.0
**G3P[4]**	2	0.2	2	0.1	1	0.1	1	0.1	6	0.1
**G3P[6]**	1	0.1	1	0.0	0	0.0	0	0.0	2	0.0
**G3P[8]**	41	3.1	30	1.0	42	2.9	25	1.7	138	1.9
**G3P[9]**	4	0.3	0	0.0	0	0.0	0	0.0	4	0.1
**G3P[11]**	1	0.1	0	0.0	0	0.0	0	0.0	1	0.0
**G4P[4]**	0	0.0	0	0.0	0	0.0	2	0.1	2	0.0
**G4P[6]**	2	0.2	1	0.0	0	0.0	0	0.0	3	0.0
**G8P[6]**	0	0.0	0	0.0	0	0.0	1	0.1	1	0.0
**G8P[8]**	3	0.2	0	0.0	0	0.0	1	0.1	4	0.1
**G9P[4]**	105	8.0	194	6.5	55	3.8	63	4.2	417	5.8
**G9P[6]**	19	1.4	13	0.4	14	1.0	13	0.9	59	0.8
**G9P[8]**	41	3.1	177	6.0	24	1.7	45	3.0	287	4.0
**G10P[6]**	1	0.1	0	0.0	0	0.0	2	0.1	3	0.0
**G10P[8]**	2	0.2	0	0.0	0	0.0	2	0.1	4	0.1
**G10P[11]**	0	0.0	12	0.4	0	0.0	0	0.0	12	0.2
**G12P[4]**	5	0.4	4	0.1	0	0.0	5	0.3	14	0.2
**G12P[6]**	114	8.7	72	2.4	30	2.1	86	5.7	302	4.2
**G12P[8]**	31	2.4	88	3.0	7	0.5	45	3.0	171	2.4
**G12P[11]**	1	0.1	2	0.1	0	0.0	4	0.3	7	0.1
**Mixed**	120	9.1	153	5.2	107	7.5	106	7.0	486	6.7
**Partially typed**	127	9.7	162	5.5	209	14.6	122	8.1	620	8.6
**Untyped**	102	7.8	177	6.0	393	27.5	50	3.3	722	10.0
**Total**	1312	100.0	2962	100.0	1429	100.0	1512	100.0	7215	100.0

The distribution of G and P types showed interesting trends during the 11 years surveillance. The proportion of G1 increased from 2005 to 2014 (78% of rotavirus genotypes during 2013–2014), but decreased subsequently to only 35.5% in 2016. G2 showed an increasing trend during 2005 to 2007 (45.5%), but decreased gradually during the subsequent years to 16.5% during 2015–2016. G3 emerged during the year 2013 (0.5%), which increased to 18.6% during 2015–2016. G9 did not show any specific trend, with the lowest being detected during 2007–2008 (9.1%), and highest during 2009–2010 (28.6%). During 2015–2016, G9 contributed to 25.8% of all rotavirus genotypes. However, G12 showed an increasing trend till 2008–2009 (3.6% during 2005–2006 to 23.7% during 2008–2009), but decreased substantially during the following years to only 3.6% during 2015–2016.

Of the genotyped samples, P[4], P[6], and P[8] contributed to 99.5% of the P types. P[8] was the most common P type (63.6%). P[8] showed an increasing trend from 2005 to 2014 (81.3% during 2013–2014), after which it decreased to 49.4% in 2016. P[4] was the second most common P type, detected in 25.3% of the genotyped samples. There was no specific trend in the distribution of P[4] during 2005 to 2016. The proportion of P[4] increased from 2005 to 2007 (46% in 2007), after which it decreased to 27% in 2009. Subsequently, the proportion increased to 43.7% in 2010. From 2010 to 2014, P[4] decreased to 10.3% of all P types, after which the proportion increased to 38.1% in 2016. P[6] was detected in 10.6% of the genotyped samples. The proportion of P[6] increased from 8.4% during 2005–2006 to nearly 18% during 2007–2009. The proportion decreased during the subsequent years to 10.6% during 2016.

**Fig. 3:**
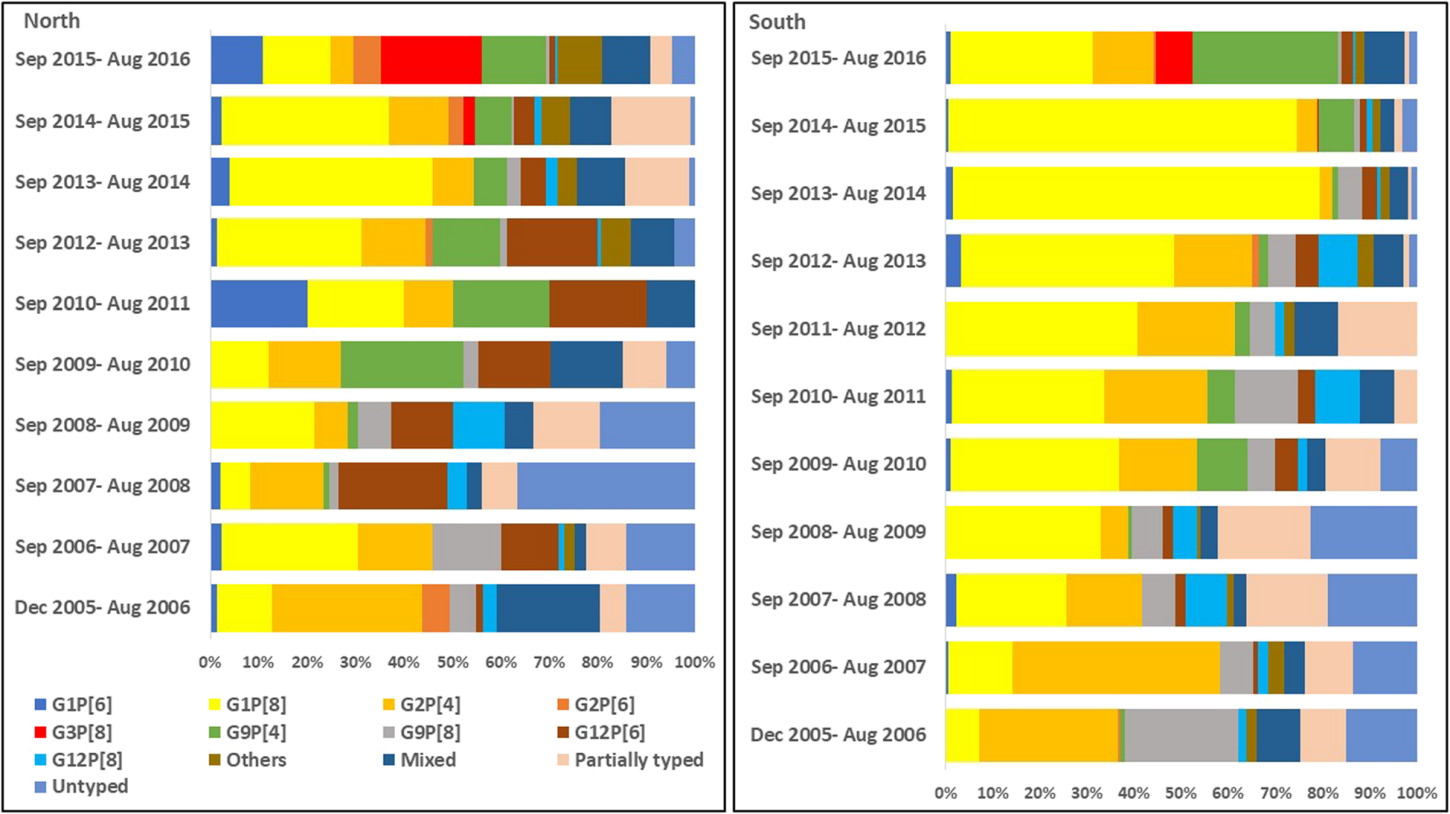
Distribution of rotavirus genotypes (G and P combination) in the northern and southern regions of India from 2005 to 2016. (“Others” includes all rotavirus genotypes with an overall proportion of < 1%)

**Fig. 4:**
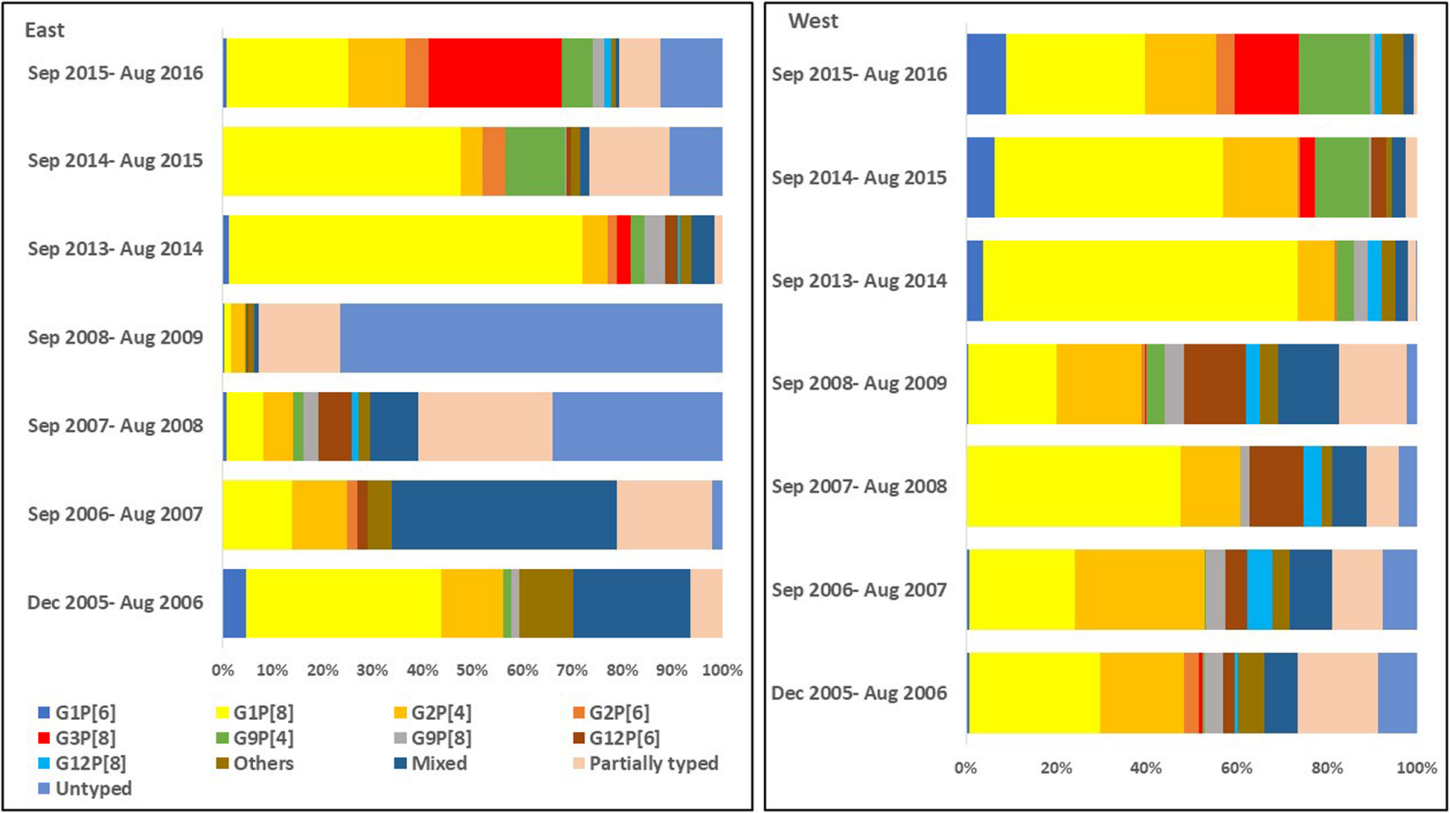
Distribution of rotavirus genotypes (G and P combination) in the eastern and western regions of India from 2005 to 2016. (“Others” includes all rotavirus genotypes with an overall proportion of < 1%)

The distribution by year of rotavirus genotypes (G and P combinations) in the four geographical regions is provided in Figs. 3 and 4 (Supplementary tables S1-S4). There were significant differences in the proportional representation of common genotypes such as G2P[4] and G9P[4] over the surveillance period. In the multinomial logistic regression model, where G1P[8] was taken as the reference category, G2P[4] showed a significantly decreasing trend over the surveillance period (adjusted multinomial odds ratio: 0.78; 95% confidence interval: 0.76–0.80; *p* < 0.001). However, G9P[4] showed a significant increase in the later years of the surveillance (adjusted multinomial OR: 1.33; 95% CI 1.25–1.41, p < 0.001).

Proportional representation of partially typed and untyped samples consistently decreased over the surveillance period (aMOR = 0.74, 95% CI 0.72–0.75, p < 0.001), probably explained by inclusion of additional approaches to reduce untyped samples in the later years of the surveillance [21].

Detection of less common genotypes (other than G1P[8], G2P[4] and G9P[4]) was significantly more common in the age group 0–11 months (aMOR = 0.80; 95% CI 0.73–0.88). Higher representation of mixed and partially typed/untyped samples was also seen more in the age group 0–11 months (mixed: aMOR = 0.86, 95% CI 0.75–0.99, *p* = 0.043; partially typed/untyped: aMOR = 0.91, 95% CI 0.82–1, *p* = 0.053).

## Discussion

Testing more than 29,000 samples over a period of 11 years from multiple sites in India demonstrated the ubiquity and high prevalence of rotavirus (37%) as a cause of AGE. These findings are consistent with previous Indian findings of approximately 34% (inter study variation: 19–50%) positivity for rotavirus diarrhoea requiring hospitalization [4, 22, 23]. Prior findings of seasonality were also confirmed, although some earlier studies have found no such association [4, 10, 24–26]. Similar to findings of our study, a multicentric surveillance study on rotavirus diarrhoea during 2004–2005 in Europe involving 7 countries (Belgium, France, Germany, Italy, Spain, Sweden, and United Kingdom) found 40.6% of acute gastroenteritis in children < 5 years to be due to rotavirus [27]. However, the proportion of rotavirus diarrhoea in children < 5 years before the introduction of rotavirus vaccine in Latin America (24.3%) and the United Sates (25.6%) were lower than that in India [28, 29].

From 2005 to 2016, there was a notable shift in the rotavirus genotypes. The G12 genotypes, particularly G12P[6] in the north and G12P[8] in the south, showed an increase till 2013 but the proportion reduced during the subsequent years. The G12 genotype was first detected in children with diarrhoea in the Philippines during 1987–1988, and was subsequently reported from Thailand and USA (1998–1999), and from India in 2003 from the eastern region [30–33]. In the following years, G12 was reported from other geographical regions of India also [20, 34], and from neighbouring countries such as Nepal, Myanmar, Bangladesh, and Sri Lanka [35–43]. During 2005 to 2013, studies on rotavirus diarrhoea in hospitalized children from different continents including Asia found that G12 positivity ranged from 10 to 86% of rotavirus gastroenteritis [44].

In this surveillance, G9 in association with P[4], P[6], and P[8] constituted 11% of the rotavirus positive samples, G9P[4] being the most common in the northern region (8%), compared to G9P[8] in the southern region (6%). G9 rotavirus genotype was first detected during 1983–1984 in Philadelphia, USA, causing diarrhoea in infants [30]. Subsequently, G9 associated with diarrhoea, as opposed to the asymptomatic G9P[11] strains from neonatal nurseries, was reported from several countries during the 1990s, including India in 1993 [30, 45]. Currently, G9 genotype (particularly G9P[8]) is one of the 6 most common genotypes globally (along with G1P[8], G2P[4], G3P[8], G4P[8], and G12P[8]), causing approximately 90% of severe rotavirus disease requiring hospitalization [46]. While the source of G9 and G12 genotypes in humans is not known for certain, there have been reports of closely related G9 and G12 genotypes in pigs, suggesting a potential porcine origin of these genotypes [47–49].

G1P[8] and G2P[4] were the two most common genotypes in this surveillance, and comprised more than 50% of the genotyped samples. These two genotypes have been commonly detected in other surveillance studies in India as well [5–7, 9–12]. A review on rotavirus infections in India found these two genotypes to cause approximately 50% of diarrhoea in non-neonates [4]. Over the surveillance period, there was a decline in the proportion of G2P[4], and an increase in the proportion of G9P[4]. A higher proportion of mixed and partially typed/untyped samples was also seen more in the age group ≤11 months. Hungerford et al. have reported similar trends with infants 0–11 months more likely than older children to be infected with mixed/untypable genotypes and less common genotypes in 7 years of surveillance across 12 European countries before the introduction of the oral rotavirus vaccine into the immunization schedule [50].

In our study, the proportion of G1P[8] decreased from 2014 onwards, which coincided with the emergence of G3P[8] genotype across all the geographical regions. Such changes in the distribution of rotavirus genotypes and the emergence of new genotypes before the introduction of rotavirus vaccine into the national immunization schedule will be important to consider while evaluating the change in rotavirus epidemiology after introduction of rotavirus vaccines. A recent review on viral gastroenteritis worldwide has found G3P[8] to be one of the six most common genotypes globally, causing 90% of the rotavirus associated diarrhoea requiring medical attention [46].

The detection of uncommon genotypes such as G1P[4], G1P[6], G2P[6], G2P[8], G3P[4], G3P[6], G10P[11], G12P[4], G12P[11], etc., and a high proportion of mixed infections (ranged from 5.2% in the south to 9.1% in the north) are an indication that children probably acquire rotavirus infections from various sources, and could serve as sources of new strains globally. Similar to our study, unusual rotavirus genotypes have been reported to cause approximately 4.9% of rotavirus diarrhoea worldwide [30]. A review on epidemiology of rotavirus in India reported that 9% of rotavirus infections are of mixed type [4]. The detection of a high proportion of untyped or non-typeable samples in our study could be due to very low number of viral particles with intact RNA in stool samples, non-recognition of the viruses by the primer sets due to point mutations at the primer binding sites, or the viruses belonging to genotypes which are not included in the primer set used in the RT-PCR assays [12, 30].

The strength of this surveillance is the use of a standardized protocol for recruitment of cases. One limitation of the study is the availability of different number of sites during the different periods of surveillance. While data from northern and southern regions is available for all the years of surveillance from 2005 to 2016 (although there was variation in the number of available sites), no data is available from the eastern and western regions from August 2009 to September 2013. This could have led to potential delay in the detection of G3P[8] in the eastern region, where it was first reported during 2013–2014 (2.7%).

## Conclusion

The study highlights the substantial burden of rotavirus gastroenteritis in Indian children < 5 years of age before the introduction of the oral rotavirus vaccine into the national immunization schedule. The study also demonstrates the diversity of circulating rotavirus genotypes causing diarrhoea in children across the different geographical regions of India, along with the emergence of new genotypes. With the introduction of the rotavirus vaccine into the national immunization program in 2016, continued surveillance will be important to evaluate the potential change in epidemiology of rotavirus gastroenteritis and the vaccine effectiveness against a broad range of genotypes.

## Supplementary information

**Additional file 1: Table S1**: Year wise distribution of rotavirus genotypes in the northern region from 2005 to 2016. The table contains the year wise distribution of rotavirus genotypes causing diarrhoea in children < 5 years of age in the northern region from 2005 to 2016.

**Additional file 2: Table S2**: Year wise distribution of rotavirus genotypes in the southern region from 2005 to 2016. The table contains the year wise distribution of rotavirus genotypes causing diarrhoea in children < 5 years of age in the southern region from 2005 to 2016.

**Additional file 3: Table S3**: Year wise distribution of rotavirus genotypes in the eastern region from 2005 to 2016. The table contains the year wise distribution of rotavirus genotypes causing diarrhoea in children < 5 years of age in the eastern region from 2005 to 2016.

**Additional file 4: Table S4**: Year wise distribution of rotavirus genotypes in the western region from 2005 to 2016. The table contains the year wise distribution of rotavirus genotypes causing diarrhoea in children < 5 years of age in the western region from 2005 to 2016.

## Data Availability

Since the study is continuing in some sites, data are still being generated and have not been placed in a public repository. The data analysed during the period reported in this manuscript will be made available on request after de-identification.

## Abbreviations

AGE: Acute gastroenteritis
EIA: Enzyme Immunoassay
RT-PCR: Reverse Transcription-Polymerase Chain Reaction
