# Supplementary material for: Diversity of rotavirus genotypes circulating in children < 5 years of age hospitalized for acute gastroenteritis in India from 2005 to 2016: analysis of temporal and regional genotype variation
Source: BMC Infect Dis. 2020 Oct 9;20:740. doi: 10.1186/s12879-020-05448-y (PMC7547507; doi:10.1186/s12879-020-05448-y)
Supplement: Supplementary file 1 — Additional file 1: Table S1: Year wise distribution of rotavirus genotypes in the northern region from 2005 to 2016. The table contains the year wise distribution of rotavirus genotypes causing diarrhoea in children < 5 years of age in the northern region from 2005 to 2016. [file 12879_2020_5448_MOESM1_ESM.docx]

**Table S1:** Year wise distribution of rotavirus genotypes in the northern region from 2005 to 2016

| **North** | **December, 2005- August, 2006** | | **September 2006- August 2007** | | **September 2007- August 2008** | | **September 2008- August 2009** | | **September 2009- August 2010** | | **September 2010- August 2011** | | **September 2012- August 2013** | | **September 2013- August 2014** | | **September 2014- August 2015** | | **September 2015- August 2016** | | **Total** | |
| --- | --- | --- | --- | --- | --- | --- | --- | --- | --- | --- | --- | --- | --- | --- | --- | --- | --- | --- | --- | --- | --- | --- |
|  | N | % | N | % | N | % | N | % | N | % | N | % | N | % | N | % | N | % | N | % | N | % |
| **G1P[4]** | 0 | 0.0 | 1 | 1.2 | 0 | 0.0 | 0 | 0.0 | 0 | 0.0 | 0 | 0.0 | 1 | 0.7 | 3 | 0.9 | 1 | 0.5 | 1 | 0.6 | 7 | 0.5 |
| **G1P[6]** | 1 | 1.4 | 2 | 2.4 | 2 | 2.0 | 0 | 0.0 | 0 | 0.0 | 2 | 20.0 | 2 | 1.4 | 14 | 4.0 | 5 | 2.3 | 19 | 11.0 | 47 | 3.6 |
| **G1P[8]** | 8 | 11.3 | 24 | 28.2 | 6 | 6.1 | 22 | 21.6 | 8 | 11.9 | 2 | 20.0 | 43 | 29.9 | 146 | 42.0 | 74 | 34.6 | 24 | 13.9 | 357 | 27.2 |
| **G1P[9]** | 0 | 0.0 | 0 | 0.0 | 0 | 0.0 | 0 | 0.0 | 0 | 0.0 | 0 | 0.0 | 0 | 0.0 | 0 | 0.0 | 0 | 0.0 | 1 | 0.6 | 1 | 0.1 |
| **G1P[11]** | 0 | 0.0 | 0 | 0.0 | 0 | 0.0 | 0 | 0.0 | 0 | 0.0 | 0 | 0.0 | 0 | 0.0 | 1 | 0.3 | 0 | 0.0 | 0 | 0.0 | 1 | 0.1 |
| **G2P[4]** | 22 | 31.0 | 13 | 15.3 | 15 | 15.3 | 7 | 6.9 | 10 | 14.9 | 1 | 10.0 | 19 | 13.2 | 29 | 8.3 | 26 | 12.1 | 8 | 4.6 | 150 | 11.4 |
| **G2P[6]** | 4 | 5.6 | 0 | 0.0 | 0 | 0.0 | 0 | 0.0 | 0 | 0.0 | 0 | 0.0 | 2 | 1.4 | 0 | 0.0 | 7 | 3.3 | 10 | 5.8 | 23 | 1.8 |
| **G2P[8]** | 0 | 0.0 | 0 | 0.0 | 0 | 0.0 | 0 | 0.0 | 0 | 0.0 | 0 | 0.0 | 0 | 0.0 | 0 | 0.0 | 3 | 1.4 | 0 | 0.0 | 3 | 0.2 |
| **G2P[10]** | 0 | 0.0 | 0 | 0.0 | 0 | 0.0 | 0 | 0.0 | 0 | 0.0 | 0 | 0.0 | 0 | 0.0 | 0 | 0.0 | 0 | 0.0 | 0 | 0.0 | 0 | 0.0 |
| **G2P[11]** | 0 | 0.0 | 0 | 0.0 | 0 | 0.0 | 0 | 0.0 | 0 | 0.0 | 0 | 0.0 | 0 | 0.0 | 1 | 0.3 | 0 | 0.0 | 0 | 0.0 | 1 | 0.1 |
| **G3P[4]** | 0 | 0.0 | 0 | 0.0 | 0 | 0.0 | 0 | 0.0 | 0 | 0.0 | 0 | 0.0 | 0 | 0.0 | 0 | 0.0 | 1 | 0.5 | 1 | 0.6 | 2 | 0.2 |
| **G3P[6]** | 0 | 0.0 | 0 | 0.0 | 0 | 0.0 | 0 | 0.0 | 0 | 0.0 | 0 | 0.0 | 0 | 0.0 | 0 | 0.0 | 1 | 0.5 | 0 | 0.0 | 1 | 0.1 |
| **G3P[8]** | 0 | 0.0 | 0 | 0.0 | 0 | 0.0 | 0 | 0.0 | 0 | 0.0 | 0 | 0.0 | 0 | 0.0 | 0 | 0.0 | 5 | 2.3 | 36 | 20.8 | 41 | 3.1 |
| **G3P[9]** | 0 | 0.0 | 0 | 0.0 | 0 | 0.0 | 0 | 0.0 | 0 | 0.0 | 0 | 0.0 | 0 | 0.0 | 0 | 0.0 | 0 | 0.0 | 4 | 2.3 | 4 | 0.3 |
| **G3P[11]** | 0 | 0.0 | 0 | 0.0 | 0 | 0.0 | 0 | 0.0 | 0 | 0.0 | 0 | 0.0 | 0 | 0.0 | 0 | 0.0 | 0 | 0.0 | 1 | 0.6 | 1 | 0.1 |
| **G4P[4]** | 0 | 0.0 | 0 | 0.0 | 0 | 0.0 | 0 | 0.0 | 0 | 0.0 | 0 | 0.0 | 0 | 0.0 | 0 | 0.0 | 0 | 0.0 | 0 | 0.0 | 0 | 0.0 |
| **G4P[6]** | 0 | 0.0 | 0 | 0.0 | 0 | 0.0 | 0 | 0.0 | 0 | 0.0 | 0 | 0.0 | 0 | 0.0 | 0 | 0.0 | 2 | 0.9 | 0 | 0.0 | 2 | 0.2 |
| **G8P[6]** | 0 | 0.0 | 0 | 0.0 | 0 | 0.0 | 0 | 0.0 | 0 | 0.0 | 0 | 0.0 | 0 | 0.0 | 0 | 0.0 | 0 | 0.0 | 0 | 0.0 | 0 | 0.0 |
| **G8P[8]** | 0 | 0.0 | 0 | 0.0 | 0 | 0.0 | 0 | 0.0 | 0 | 0.0 | 0 | 0.0 | 0 | 0.0 | 0 | 0.0 | 1 | 0.5 | 2 | 1.2 | 3 | 0.2 |
| **G9P[4]** | 0 | 0.0 | 0 | 0.0 | 1 | 1.0 | 2 | 2.0 | 17 | 25.4 | 2 | 20.0 | 20 | 13.9 | 24 | 6.9 | 16 | 7.5 | 23 | 13.3 | 105 | 8.0 |
| **G9P[6]** | 0 | 0.0 | 1 | 1.2 | 0 | 0.0 | 0 | 0.0 | 0 | 0.0 | 0 | 0.0 | 5 | 3.5 | 9 | 2.6 | 3 | 1.4 | 1 | 0.6 | 19 | 1.4 |
| **G9P[8]** | 4 | 5.6 | 12 | 14.1 | 2 | 2.0 | 7 | 6.9 | 2 | 3.0 | 0 | 0.0 | 2 | 1.4 | 10 | 2.9 | 1 | 0.5 | 1 | 0.6 | 41 | 3.1 |
| **G10P[6]** | 0 | 0.0 | 0 | 0.0 | 0 | 0.0 | 0 | 0.0 | 0 | 0.0 | 0 | 0.0 | 0 | 0.0 | 0 | 0.0 | 0 | 0.0 | 1 | 0.6 | 1 | 0.1 |
| **G10P[8]** | 0 | 0.0 | 0 | 0.0 | 0 | 0.0 | 0 | 0.0 | 0 | 0.0 | 0 | 0.0 | 0 | 0.0 | 0 | 0.0 | 0 | 0.0 | 2 | 1.2 | 2 | 0.2 |
| **G10P[11]** | 0 | 0.0 | 0 | 0.0 | 0 | 0.0 | 0 | 0.0 | 0 | 0.0 | 0 | 0.0 | 0 | 0.0 | 0 | 0.0 | 0 | 0.0 | 0 | 0.0 | 0 | 0.0 |
| **G12P[4]** | 0 | 0.0 | 0 | 0.0 | 0 | 0.0 | 0 | 0.0 | 0 | 0.0 | 0 | 0.0 | 3 | 2.1 | 0 | 0.0 | 1 | 0.5 | 1 | 0.6 | 5 | 0.4 |
| **G12P[6]** | 1 | 1.4 | 10 | 11.8 | 22 | 22.4 | 13 | 12.7 | 10 | 14.9 | 2 | 20.0 | 27 | 18.8 | 18 | 5.2 | 9 | 4.2 | 2 | 1.2 | 114 | 8.7 |
| **G12P[8]** | 2 | 2.8 | 1 | 1.2 | 4 | 4.1 | 11 | 10.8 | 0 | 0.0 | 0 | 0.0 | 1 | 0.7 | 8 | 2.3 | 3 | 1.4 | 1 | 0.6 | 31 | 2.4 |
| **G12P[11]** | 0 | 0.0 | 0 | 0.0 | 0 | 0.0 | 0 | 0.0 | 0 | 0.0 | 0 | 0.0 | 0 | 0.0 | 0 | 0.0 | 0 | 0.0 | 1 | 0.6 | 1 | 0.1 |
| **Mixed** | 15 | 21.1 | 2 | 2.4 | 3 | 3.1 | 6 | 5.9 | 10 | 14.9 | 1 | 10.0 | 13 | 9.0 | 35 | 10.1 | 18 | 8.4 | 17 | 9.8 | 120 | 9.1 |
| **Partially typed** | 4 | 5.6 | 7 | 8.2 | 7 | 7.1 | 14 | 13.7 | 6 | 9.0 | 0 | 0.0 | 0 | 0.0 | 46 | 13.2 | 35 | 16.4 | 8 | 4.6 | 127 | 9.7 |
| **Untyped** | 10 | 14.1 | 12 | 14.1 | 36 | 36.7 | 20 | 19.6 | 4 | 6.0 | 0 | 0.0 | 6 | 4.2 | 4 | 1.1 | 2 | 0.9 | 8 | 4.6 | 102 | 7.8 |
| **Total** | 71 | 100.0 | 85 | 100.0 | 98 | 100.0 | 102 | 100.0 | 67 | 100.0 | 10 | 100.0 | 144 | 100.0 | 348 | 100.0 | 214 | 100.0 | 173 | 100.0 | 1312 | 100.0 |
