# Supplementary material for: Diversity of rotavirus genotypes circulating in children < 5 years of age hospitalized for acute gastroenteritis in India from 2005 to 2016: analysis of temporal and regional genotype variation
Source: BMC Infect Dis. 2020 Oct 9;20:740. doi: 10.1186/s12879-020-05448-y (PMC7547507; doi:10.1186/s12879-020-05448-y)
Supplement: Supplementary file 2 — Additional file 2: Table S2: Year wise distribution of rotavirus genotypes in the southern region from 2005 to 2016. The table contains the year wise distribution of rotavirus genotypes causing diarrhoea in children < 5 years of age in the southern region from 2005 to 2016. [file 12879_2020_5448_MOESM2_ESM.docx]

**Table S2:** Year wise distribution of rotavirus genotypes in the southern region from 2005 to 2016

| **South** | **December, 2005- August, 2006** | | **September 2006- August 2007** | | **September 2007- August 2008** | | **September 2008- August 2009** | | **September 2009- August 2010** | | **September 2010- August 2011** | | **September 2011- August 2012** | | **September 2012- August 2013** | | **September 2013- August 2014** | | **September 2014- August 2015** | | **September 2015- August 2016** | | **Total** |  |
| --- | --- | --- | --- | --- | --- | --- | --- | --- | --- | --- | --- | --- | --- | --- | --- | --- | --- | --- | --- | --- | --- | --- | --- | --- |
|  | **N** | **%** | **N** | **%** | **N** | **%** | **N** | **%** | **N** | **%** | **N** | **%** | **N** | **%** | **N** | **%** | **N** | **%** | **N** | **%** | **N** | **%** | **N** | **%** |
| **G1P[4]** | 0 | 0.0 | 0 | 0.0 | 0 | 0.0 | 1 | 0.7 | 0 | 0.0 | 0 | 0.0 | 2 | 2.1 | 5 | 1.4 | 6 | 0.9 | 3 | 0.6 | 0 | 0.0 | 17 | 0.6 |
| **G1P[6]** | 0 | 0.0 | 1 | 0.5 | 5 | 2.2 | 0 | 0.0 | 1 | 1.0 | 1 | 1.2 | 0 | 0.0 | 11 | 3.2 | 10 | 1.5 | 3 | 0.6 | 4 | 1.1 | 36 | 1.2 |
| **G1P[8]** | 16 | 7.0 | 25 | 13.6 | 53 | 23.5 | 45 | 32.8 | 37 | 35.9 | 27 | 32.5 | 39 | 40.6 | 157 | 45.4 | 510 | 77.7 | 391 | 73.9 | 113 | 30.1 | 1413 | 47.7 |
| **G1P[9]** | 0 | 0.0 | 0 | 0.0 | 0 | 0.0 | 0 | 0.0 | 0 | 0.0 | 0 | 0.0 | 0 | 0.0 | 0 | 0.0 | 0 | 0.0 | 0 | 0.0 | 0 | 0.0 | 0 | 0.0 |
| **G1P[11]** | 0 | 0.0 | 0 | 0.0 | 0 | 0.0 | 0 | 0.0 | 0 | 0.0 | 0 | 0.0 | 0 | 0.0 | 0 | 0.0 | 0 | 0.0 | 0 | 0.0 | 0 | 0.0 | 0 | 0.0 |
| **G2P[4]** | 67 | 29.5 | 81 | 44.0 | 36 | 15.9 | 8 | 5.8 | 17 | 16.5 | 18 | 21.7 | 20 | 20.8 | 57 | 16.5 | 19 | 2.9 | 23 | 4.3 | 48 | 12.8 | 394 | 13.3 |
| **G2P[6]** | 1 | 0.4 | 0 | 0.0 | 0 | 0.0 | 0 | 0.0 | 0 | 0.0 | 0 | 0.0 | 0 | 0.0 | 5 | 1.4 | 0 | 0.0 | 0 | 0.0 | 2 | 0.5 | 8 | 0.3 |
| **G2P[8]** | 2 | 0.9 | 3 | 1.6 | 0 | 0.0 | 0 | 0.0 | 0 | 0.0 | 0 | 0.0 | 0 | 0.0 | 0 | 0.0 | 1 | 0.2 | 0 | 0.0 | 0 | 0.0 | 6 | 0.2 |
| **G2P[10]** | 0 | 0.0 | 0 | 0.0 | 0 | 0.0 | 0 | 0.0 | 0 | 0.0 | 0 | 0.0 | 0 | 0.0 | 0 | 0.0 | 0 | 0.0 | 0 | 0.0 | 0 | 0.0 | 0 | 0.0 |
| **G2P[11]** | 0 | 0.0 | 0 | 0.0 | 0 | 0.0 | 0 | 0.0 | 0 | 0.0 | 0 | 0.0 | 0 | 0.0 | 0 | 0.0 | 0 | 0.0 | 0 | 0.0 | 0 | 0.0 | 0 | 0.0 |
| **G3P[4]** | 0 | 0.0 | 0 | 0.0 | 0 | 0.0 | 0 | 0.0 | 0 | 0.0 | 0 | 0.0 | 0 | 0.0 | 0 | 0.0 | 0 | 0.0 | 0 | 0.0 | 2 | 0.5 | 2 | 0.1 |
| **G3P[6]** | 0 | 0.0 | 0 | 0.0 | 0 | 0.0 | 0 | 0.0 | 0 | 0.0 | 0 | 0.0 | 0 | 0.0 | 0 | 0.0 | 0 | 0.0 | 1 | 0.2 | 0 | 0.0 | 1 | 0.0 |
| **G3P[8]** | 0 | 0.0 | 0 | 0.0 | 0 | 0.0 | 0 | 0.0 | 0 | 0.0 | 0 | 0.0 | 0 | 0.0 | 0 | 0.0 | 0 | 0.0 | 1 | 0.2 | 29 | 7.7 | 30 | 1.0 |
| **G3P[9]** | 0 | 0.0 | 0 | 0.0 | 0 | 0.0 | 0 | 0.0 | 0 | 0.0 | 0 | 0.0 | 0 | 0.0 | 0 | 0.0 | 0 | 0.0 | 0 | 0.0 | 0 | 0.0 | 0 | 0.0 |
| **G3P[11]** | 0 | 0.0 | 0 | 0.0 | 0 | 0.0 | 0 | 0.0 | 0 | 0.0 | 0 | 0.0 | 0 | 0.0 | 0 | 0.0 | 0 | 0.0 | 0 | 0.0 | 0 | 0.0 | 0 | 0.0 |
| **G4P[4]** | 0 | 0.0 | 0 | 0.0 | 0 | 0.0 | 0 | 0.0 | 0 | 0.0 | 0 | 0.0 | 0 | 0.0 | 0 | 0.0 | 0 | 0.0 | 0 | 0.0 | 0 | 0.0 | 0 | 0.0 |
| **G4P[6]** | 0 | 0.0 | 0 | 0.0 | 0 | 0.0 | 0 | 0.0 | 0 | 0.0 | 0 | 0.0 | 0 | 0.0 | 0 | 0.0 | 0 | 0.0 | 0 | 0.0 | 1 | 0.3 | 1 | 0.0 |
| **G8P[6]** | 0 | 0.0 | 0 | 0.0 | 0 | 0.0 | 0 | 0.0 | 0 | 0.0 | 0 | 0.0 | 0 | 0.0 | 0 | 0.0 | 0 | 0.0 | 0 | 0.0 | 0 | 0.0 | 0 | 0.0 |
| **G8P[8]** | 0 | 0.0 | 0 | 0.0 | 0 | 0.0 | 0 | 0.0 | 0 | 0.0 | 0 | 0.0 | 0 | 0.0 | 0 | 0.0 | 0 | 0.0 | 0 | 0.0 | 0 | 0.0 | 0 | 0.0 |
| **G9P[4]** | 2 | 0.9 | 0 | 0.0 | 0 | 0.0 | 1 | 0.7 | 11 | 10.7 | 5 | 6.0 | 3 | 3.1 | 7 | 2.0 | 8 | 1.2 | 41 | 7.8 | 116 | 30.9 | 194 | 6.5 |
| **G9P[6]** | 2 | 0.9 | 2 | 1.1 | 2 | 0.9 | 0 | 0.0 | 0 | 0.0 | 0 | 0.0 | 0 | 0.0 | 1 | 0.3 | 5 | 0.8 | 1 | 0.2 | 0 | 0.0 | 13 | 0.4 |
| **G9P[8]** | 55 | 24.2 | 13 | 7.1 | 16 | 7.1 | 9 | 6.6 | 6 | 5.8 | 11 | 13.3 | 5 | 5.2 | 20 | 5.8 | 33 | 5.0 | 6 | 1.1 | 3 | 0.8 | 177 | 6.0 |
| **G10P[6]** | 0 | 0.0 | 0 | 0.0 | 0 | 0.0 | 0 | 0.0 | 0 | 0.0 | 0 | 0.0 | 0 | 0.0 | 0 | 0.0 | 0 | 0.0 | 0 | 0.0 | 0 | 0.0 | 0 | 0.0 |
| **G10P[8]** | 0 | 0.0 | 0 | 0.0 | 0 | 0.0 | 0 | 0.0 | 0 | 0.0 | 0 | 0.0 | 0 | 0.0 | 0 | 0.0 | 0 | 0.0 | 0 | 0.0 | 0 | 0.0 | 0 | 0.0 |
| **G10P[11]** | 1 | 0.4 | 0 | 0.0 | 1 | 0.4 | 0 | 0.0 | 0 | 0.0 | 0 | 0.0 | 0 | 0.0 | 3 | 0.9 | 1 | 0.2 | 4 | 0.8 | 2 | 0.5 | 12 | 0.4 |
| **G12P[4]** | 0 | 0.0 | 1 | 0.5 | 0 | 0.0 | 0 | 0.0 | 0 | 0.0 | 0 | 0.0 | 0 | 0.0 | 3 | 0.9 | 0 | 0.0 | 0 | 0.0 | 0 | 0.0 | 4 | 0.1 |
| **G12P[6]** | 0 | 0.0 | 2 | 1.1 | 5 | 2.2 | 3 | 2.2 | 5 | 4.9 | 3 | 3.6 | 0 | 0.0 | 17 | 4.9 | 20 | 3.0 | 8 | 1.5 | 9 | 2.4 | 72 | 2.4 |
| **G12P[8]** | 4 | 1.8 | 4 | 2.2 | 20 | 8.8 | 7 | 5.1 | 2 | 1.9 | 8 | 9.6 | 2 | 2.1 | 28 | 8.1 | 5 | 0.8 | 6 | 1.1 | 2 | 0.5 | 88 | 3.0 |
| **G12P[11]** | 0 | 0.0 | 0 | 0.0 | 0 | 0.0 | 0 | 0.0 | 0 | 0.0 | 0 | 0.0 | 0 | 0.0 | 0 | 0.0 | 0 | 0.0 | 0 | 0.0 | 2 | 0.5 | 2 | 0.1 |
| **Mixed** | 21 | 9.3 | 8 | 4.3 | 6 | 2.7 | 5 | 3.6 | 4 | 3.9 | 6 | 7.2 | 9 | 9.4 | 22 | 6.4 | 25 | 3.8 | 15 | 2.8 | 32 | 8.5 | 153 | 5.2 |
| **Partially typed** | 22 | 9.7 | 19 | 10.3 | 39 | 17.3 | 27 | 19.7 | 12 | 11.7 | 4 | 4.8 | 16 | 16.7 | 4 | 1.2 | 5 | 0.8 | 10 | 1.9 | 4 | 1.1 | 162 | 5.5 |
| **Untyped** | 34 | 15.0 | 25 | 13.6 | 43 | 19.0 | 31 | 22.6 | 8 | 7.8 | 0 | 0.0 | 0 | 0.0 | 6 | 1.7 | 8 | 1.2 | 16 | 3.0 | 6 | 1.6 | 177 | 6.0 |
| **Total** | 227 | 100.0 | 184 | 100.0 | 226 | 100.0 | 137 | 100.0 | 103 | 100.0 | 83 | 100.0 | 96 | 100.0 | 346 | 100.0 | 656 | 100.0 | 529 | 100.0 | 375 | 100.0 | 2962 | 100.0 |
