# Supplementary material for: Diversity of rotavirus genotypes circulating in children < 5 years of age hospitalized for acute gastroenteritis in India from 2005 to 2016: analysis of temporal and regional genotype variation
Source: BMC Infect Dis. 2020 Oct 9;20:740. doi: 10.1186/s12879-020-05448-y (PMC7547507; doi:10.1186/s12879-020-05448-y)
Supplement: Supplementary file 3 — Additional file 3: Table S3: Year wise distribution of rotavirus genotypes in the eastern region from 2005 to 2016. The table contains the year wise distribution of rotavirus genotypes causing diarrhoea in children < 5 years of age in the eastern region from 2005 to 2016. [file 12879_2020_5448_MOESM3_ESM.docx]

**Table S3:** Year wise distribution of rotavirus genotypes in the eastern region from 2005 to 2016

| **East** | **December, 2005- August, 2006** | | **September 2006- August 2007** | | **September 2007- August 2008** | | **September 2008- August 2009** | | **September 2013- August 2014** | | **September 2014- August 2015** | | **September 2015- August 2016** | | **Total** | |
| --- | --- | --- | --- | --- | --- | --- | --- | --- | --- | --- | --- | --- | --- | --- | --- | --- |
|  | **N** | **%** | **N** | **%** | **N** | **%** | **N** | **%** | **N** | **%** | **N** | **%** | **N** | **%** | **N** | **%** |
| **G1P[4]** | 2 | 3.1 | 4 | 4.0 | 0 | 0.0 | 0 | 0.0 | 0 | 0.0 | 2 | 0.7 | 0 | 0.0 | 8 | 0.6 |
| **G1P[6]** | 3 | 4.7 | 0 | 0.0 | 2 | 0.7 | 1 | 0.3 | 3 | 1.1 | 0 | 0.0 | 1 | 0.8 | 10 | 0.7 |
| **G1P[8]** | 25 | 39.1 | 14 | 14.0 | 20 | 7.4 | 5 | 1.5 | 186 | 71.0 | 127 | 47.6 | 32 | 24.4 | 409 | 28.6 |
| **G1P[9]** | 0 | 0.0 | 0 | 0.0 | 0 | 0.0 | 1 | 0.3 | 0 | 0.0 | 0 | 0.0 | 0 | 0.0 | 1 | 0.1 |
| **G1P[11]** | 0 | 0.0 | 0 | 0.0 | 1 | 0.4 | 0 | 0.0 | 0 | 0.0 | 0 | 0.0 | 0 | 0.0 | 1 | 0.1 |
| **G2P[4]** | 8 | 12.5 | 11 | 11.0 | 16 | 5.9 | 9 | 2.7 | 13 | 5.0 | 12 | 4.5 | 15 | 11.5 | 84 | 5.9 |
| **G2P[6]** | 0 | 0.0 | 2 | 2.0 | 0 | 0.0 | 0 | 0.0 | 5 | 1.9 | 12 | 4.5 | 6 | 4.6 | 25 | 1.7 |
| **G2P[8]** | 1 | 1.6 | 1 | 1.0 | 3 | 1.1 | 0 | 0.0 | 3 | 1.1 | 0 | 0.0 | 0 | 0.0 | 8 | 0.6 |
| **G2P[10]** | 0 | 0.0 | 0 | 0.0 | 0 | 0.0 | 0 | 0.0 | 0 | 0.0 | 0 | 0.0 | 0 | 0.0 | 0 | 0.0 |
| **G2P[11]** | 0 | 0.0 | 0 | 0.0 | 0 | 0.0 | 0 | 0.0 | 0 | 0.0 | 1 | 0.4 | 0 | 0.0 | 1 | 0.1 |
| **G3P[4]** | 0 | 0.0 | 0 | 0.0 | 0 | 0.0 | 0 | 0.0 | 0 | 0.0 | 0 | 0.0 | 1 | 0.8 | 1 | 0.1 |
| **G3P[6]** | 0 | 0.0 | 0 | 0.0 | 0 | 0.0 | 0 | 0.0 | 0 | 0.0 | 0 | 0.0 | 0 | 0.0 | 0 | 0.0 |
| **G3P[8]** | 0 | 0.0 | 0 | 0.0 | 0 | 0.0 | 0 | 0.0 | 7 | 2.7 | 0 | 0.0 | 35 | 26.7 | 42 | 2.9 |
| **G3P[9]** | 0 | 0.0 | 0 | 0.0 | 0 | 0.0 | 0 | 0.0 | 0 | 0.0 | 0 | 0.0 | 0 | 0.0 | 0 | 0.0 |
| **G3P[11]** | 0 | 0.0 | 0 | 0.0 | 0 | 0.0 | 0 | 0.0 | 0 | 0.0 | 0 | 0.0 | 0 | 0.0 | 0 | 0.0 |
| **G4P[4]** | 0 | 0.0 | 0 | 0.0 | 0 | 0.0 | 0 | 0.0 | 0 | 0.0 | 0 | 0.0 | 0 | 0.0 | 0 | 0.0 |
| **G4P[6]** | 0 | 0.0 | 0 | 0.0 | 0 | 0.0 | 0 | 0.0 | 0 | 0.0 | 0 | 0.0 | 0 | 0.0 | 0 | 0.0 |
| **G8P[6]** | 0 | 0.0 | 0 | 0.0 | 0 | 0.0 | 0 | 0.0 | 0 | 0.0 | 0 | 0.0 | 0 | 0.0 | 0 | 0.0 |
| **G8P[8]** | 0 | 0.0 | 0 | 0.0 | 0 | 0.0 | 0 | 0.0 | 0 | 0.0 | 0 | 0.0 | 0 | 0.0 | 0 | 0.0 |
| **G9P[4]** | 1 | 1.6 | 0 | 0.0 | 6 | 2.2 | 1 | 0.3 | 7 | 2.7 | 32 | 12.0 | 8 | 6.1 | 55 | 3.8 |
| **G9P[6]** | 4 | 6.3 | 0 | 0.0 | 2 | 0.7 | 3 | 0.9 | 3 | 1.1 | 2 | 0.7 | 0 | 0.0 | 14 | 1.0 |
| **G9P[8]** | 1 | 1.6 | 0 | 0.0 | 8 | 3.0 | 0 | 0.0 | 11 | 4.2 | 1 | 0.4 | 3 | 2.3 | 24 | 1.7 |
| **G10P[6]** | 0 | 0.0 | 0 | 0.0 | 0 | 0.0 | 0 | 0.0 | 0 | 0.0 | 0 | 0.0 | 0 | 0.0 | 0 | 0.0 |
| **G10P[8]** | 0 | 0.0 | 0 | 0.0 | 0 | 0.0 | 0 | 0.0 | 0 | 0.0 | 0 | 0.0 | 0 | 0.0 | 0 | 0.0 |
| **G10P[11]** | 0 | 0.0 | 0 | 0.0 | 0 | 0.0 | 0 | 0.0 | 0 | 0.0 | 0 | 0.0 | 0 | 0.0 | 0 | 0.0 |
| **G12P[4]** | 0 | 0.0 | 0 | 0.0 | 0 | 0.0 | 0 | 0.0 | 0 | 0.0 | 0 | 0.0 | 0 | 0.0 | 0 | 0.0 |
| **G12P[6]** | 0 | 0.0 | 2 | 2.0 | 18 | 6.6 | 1 | 0.3 | 7 | 2.7 | 2 | 0.7 | 0 | 0.0 | 30 | 2.1 |
| **G12P[8]** | 0 | 0.0 | 0 | 0.0 | 4 | 1.5 | 0 | 0.0 | 1 | 0.4 | 0 | 0.0 | 2 | 1.5 | 7 | 0.5 |
| **G12P[11]** | 0 | 0.0 | 0 | 0.0 | 0 | 0.0 | 0 | 0.0 | 0 | 0.0 | 0 | 0.0 | 0 | 0.0 | 0 | 0.0 |
| **Mixed** | 15 | 23.4 | 45 | 45.0 | 26 | 9.6 | 3 | 0.9 | 12 | 4.6 | 5 | 1.9 | 1 | 0.8 | 107 | 7.5 |
| **Partially typed** | 4 | 6.3 | 19 | 19.0 | 73 | 26.9 | 55 | 16.5 | 4 | 1.5 | 43 | 16.1 | 11 | 8.4 | 209 | 14.6 |
| **Untyped** | 0 | 0.0 | 2 | 2.0 | 92 | 33.9 | 255 | 76.3 | 0 | 0.0 | 28 | 10.5 | 16 | 12.2 | 393 | 27.5 |
| **Total** | 64 | 100.0 | 100 | 100.0 | 271 | 100.0 | 334 | 100.0 | 262 | 100.0 | 267 | 100.0 | 131 | 100.0 | 1429 | 100.0 |
