# Supplementary material for: Diversity of rotavirus genotypes circulating in children < 5 years of age hospitalized for acute gastroenteritis in India from 2005 to 2016: analysis of temporal and regional genotype variation
Source: BMC Infect Dis. 2020 Oct 9;20:740. doi: 10.1186/s12879-020-05448-y (PMC7547507; doi:10.1186/s12879-020-05448-y)
Supplement: Supplementary file 4 — Additional file 4: Table S4: Year wise distribution of rotavirus genotypes in the western region from 2005 to 2016. The table contains the year wise distribution of rotavirus genotypes causing diarrhoea in children < 5 years of age in the western region from 2005 to 2016. [file 12879_2020_5448_MOESM4_ESM.docx]

**Table S4:** Year wise distribution of rotavirus genotypes in the western region from 2005 to 2016

| **West** | **December, 2005- August, 2006** | | **September 2006- August 2007** | | **September 2007- August 2008** | | **September 2008- August 2009** | | **September 2013- August 2014** | | **September 2014- August 2015** | | **September 2015- August 2016** | | **Total** |  |
| --- | --- | --- | --- | --- | --- | --- | --- | --- | --- | --- | --- | --- | --- | --- | --- | --- |
|  | **N** | **%** | **N** | **%** | **N** | **%** | **N** | **%** | **N** | **%** | **N** | **%** | **N** | **%** | **N** | **%** |
| **G1P[4]** | 1 | 0.7 | 2 | 0.8 | 1 | 0.4 | 1 | 0.4 | 0 | 0.0 | 0 | 0.0 | 0 | 0.0 | 5 | 0.3 |
| **G1P[6]** | 1 | 0.7 | 2 | 0.8 | 0 | 0.0 | 1 | 0.4 | 12 | 3.8 | 10 | 6.3 | 11 | 8.7 | 37 | 2.4 |
| **G1P[8]** | 44 | 29.1 | 62 | 23.4 | 119 | 47.6 | 49 | 19.6 | 217 | 69.6 | 80 | 50.6 | 39 | 31.0 | 610 | 40.3 |
| **G1P[9]** | 0 | 0.0 | 0 | 0.0 | 0 | 0.0 | 0 | 0.0 | 0 | 0.0 | 0 | 0.0 | 0 | 0.0 | 0 | 0.0 |
| **G1P[11]** | 0 | 0.0 | 0 | 0.0 | 0 | 0.0 | 0 | 0.0 | 0 | 0.0 | 0 | 0.0 | 0 | 0.0 | 0 | 0.0 |
| **G2P[4]** | 28 | 18.5 | 76 | 28.7 | 33 | 13.2 | 47 | 18.8 | 26 | 8.3 | 26 | 16.5 | 20 | 15.9 | 256 | 16.9 |
| **G2P[6]** | 5 | 3.3 | 0 | 0.0 | 0 | 0.0 | 2 | 0.8 | 1 | 0.3 | 1 | 0.6 | 5 | 4.0 | 14 | 0.9 |
| **G2P[8]** | 3 | 2.0 | 4 | 1.5 | 2 | 0.8 | 6 | 2.4 | 0 | 0.0 | 0 | 0.0 | 0 | 0.0 | 15 | 1.0 |
| **G2P[10]** | 2 | 1.3 | 0 | 0.0 | 0 | 0.0 | 0 | 0.0 | 0 | 0.0 | 0 | 0.0 | 0 | 0.0 | 2 | 0.1 |
| **G2P[11]** | 0 | 0.0 | 0 | 0.0 | 0 | 0.0 | 0 | 0.0 | 0 | 0.0 | 0 | 0.0 | 0 | 0.0 | 0 | 0.0 |
| **G3P[4]** | 0 | 0.0 | 0 | 0.0 | 0 | 0.0 | 0 | 0.0 | 0 | 0.0 | 0 | 0.0 | 1 | 0.8 | 1 | 0.1 |
| **G3P[6]** | 0 | 0.0 | 0 | 0.0 | 0 | 0.0 | 0 | 0.0 | 0 | 0.0 | 0 | 0.0 | 0 | 0.0 | 0 | 0.0 |
| **G3P[8]** | 1 | 0.7 | 0 | 0.0 | 0 | 0.0 | 1 | 0.4 | 0 | 0.0 | 5 | 3.2 | 18 | 14.3 | 25 | 1.7 |
| **G3P[9]** | 0 | 0.0 | 0 | 0.0 | 0 | 0.0 | 0 | 0.0 | 0 | 0.0 | 0 | 0.0 | 0 | 0.0 | 0 | 0.0 |
| **G3P[11]** | 0 | 0.0 | 0 | 0.0 | 0 | 0.0 | 0 | 0.0 | 0 | 0.0 | 0 | 0.0 | 0 | 0.0 | 0 | 0.0 |
| **G4P[4]** | 1 | 0.7 | 0 | 0.0 | 0 | 0.0 | 1 | 0.4 | 0 | 0.0 | 0 | 0.0 | 0 | 0.0 | 2 | 0.1 |
| **G4P[6]** | 0 | 0.0 | 0 | 0.0 | 0 | 0.0 | 0 | 0.0 | 0 | 0.0 | 0 | 0.0 | 0 | 0.0 | 0 | 0.0 |
| **G8P[6]** | 0 | 0.0 | 0 | 0.0 | 0 | 0.0 | 0 | 0.0 | 1 | 0.3 | 0 | 0.0 | 0 | 0.0 | 1 | 0.1 |
| **G8P[8]** | 0 | 0.0 | 0 | 0.0 | 0 | 0.0 | 0 | 0.0 | 1 | 0.3 | 0 | 0.0 | 0 | 0.0 | 1 | 0.1 |
| **G9P[4]** | 1 | 0.7 | 1 | 0.4 | 0 | 0.0 | 10 | 4.0 | 12 | 3.8 | 19 | 12.0 | 20 | 15.9 | 63 | 4.2 |
| **G9P[6]** | 0 | 0.0 | 1 | 0.4 | 1 | 0.4 | 1 | 0.4 | 8 | 2.6 | 0 | 0.0 | 2 | 1.6 | 13 | 0.9 |
| **G9P[8]** | 6 | 4.0 | 11 | 4.2 | 5 | 2.0 | 11 | 4.4 | 10 | 3.2 | 1 | 0.6 | 1 | 0.8 | 45 | 3.0 |
| **G10P[6]** | 2 | 1.3 | 0 | 0.0 | 0 | 0.0 | 0 | 0.0 | 0 | 0.0 | 0 | 0.0 | 0 | 0.0 | 2 | 0.1 |
| **G10P[8]** | 0 | 0.0 | 0 | 0.0 | 2 | 0.8 | 0 | 0.0 | 0 | 0.0 | 0 | 0.0 | 0 | 0.0 | 2 | 0.1 |
| **G10P[11]** | 0 | 0.0 | 0 | 0.0 | 0 | 0.0 | 0 | 0.0 | 0 | 0.0 | 0 | 0.0 | 0 | 0.0 | 0 | 0.0 |
| **G12P[4]** | 0 | 0.0 | 3 | 1.1 | 0 | 0.0 | 1 | 0.4 | 0 | 0.0 | 0 | 0.0 | 1 | 0.8 | 5 | 0.3 |
| **G12P[6]** | 4 | 2.6 | 13 | 4.9 | 30 | 12.0 | 34 | 13.6 | 0 | 0.0 | 5 | 3.2 | 0 | 0.0 | 86 | 5.7 |
| **G12P[8]** | 1 | 0.7 | 15 | 5.7 | 10 | 4.0 | 8 | 3.2 | 9 | 2.9 | 0 | 0.0 | 2 | 1.6 | 45 | 3.0 |
| **G12P[11]** | 0 | 0.0 | 0 | 0.0 | 0 | 0.0 | 0 | 0.0 | 0 | 0.0 | 2 | 1.3 | 2 | 1.6 | 4 | 0.3 |
| **Mixed** | 11 | 7.3 | 25 | 9.4 | 19 | 7.6 | 34 | 13.6 | 9 | 2.9 | 5 | 3.2 | 3 | 2.4 | 106 | 7.0 |
| **Partially typed** | 27 | 17.9 | 30 | 11.3 | 18 | 7.2 | 37 | 14.8 | 5 | 1.6 | 4 | 2.5 | 1 | 0.8 | 122 | 8.1 |
| **Untyped** | 13 | 8.6 | 20 | 7.5 | 10 | 4.0 | 6 | 2.4 | 1 | 0.3 | 0 | 0.0 | 0 | 0.0 | 50 | 3.3 |
| **Total** | 151 | 100.0 | 265 | 100.0 | 250 | 100.0 | 250 | 100.0 | 312 | 100.0 | 158 | 100.0 | 126 | 100.0 | 1512 | 100.0 |
